# Towards the Generation of B-Cell Receptor Retrogenic Mice

**DOI:** 10.1371/journal.pone.0109199

**Published:** 2014-10-08

**Authors:** Jenny Freitag, Sylvia Heink, Edith Roth, Jürgen Wittmann, Hans-Martin Jäck, Thomas Kamradt

**Affiliations:** 1 Department of Immunology, University Hospital Jena, Jena, Germany; 2 Division of Molecular Immunology, Department of Internal Medicine III, Nikolaus-Fiebiger-Center, University of Erlangen-Nürnberg, Erlangen, Germany; University Medical Center of the Johannes Gutenberg University of Mainz, Germany

## Abstract

Transgenic expression of B- and T-cell receptors (BCRs and TCRs, respectively) has been a standard tool to study lymphocyte development and function *in vivo*. The generation of transgenic mice is time-consuming and, therefore, a faster method to study the biology of defined lymphocyte receptors *in vivo* would be highly welcome. Using 2A peptide-linked multicistronic retroviral vectors to transduce stem cells, TCRs can be expressed rapidly in mice of any background. We aimed at adopting this *retrogenic* technology to the *in vivo* expression of BCRs. Using a well characterised BCR specific for hen egg lysozyme (HEL), we achieved surface expression of the retrogenically encoded BCR in a *Rag*-deficient pro B-cell line *in vitro*. *In vivo*, retrogenic BCRs were detectable only intracellularly but not on the surface of B cells from wild type or *Rag*2-deficient mice. This data, together with the fact that no BCR retrogenic mouse model has been published in the 7 years since the method was originally published for TCRs, strongly suggests that achieving BCR-expression *in vivo* with retrogenic technology is highly challenging if not impossible.

## Introduction

Over the last three decades transgenic mice have been valuable tools to study the biology of lymphocytes. Prominent examples include mice that expressing transgenic B-cell receptor (BCR) recognising (neo) self-antigens, which served to identify tolerance mechanisms in B cells [1]–[3].

Breeding transgenic mice onto different backgrounds either by conventional back-crossing or the speed congenic approach is time consuming and expensive. To overcome these major limitations, a new technique to express TCRα and TCRβ chains from a 2A peptide-linked bicistronic retroviral vector using retroviral-mediated stem cell gene transfer was developed and published in 2006 [4]–[6]. These mice were designated ‘*retrogenic*’ (‘*retro*’ from retrovirus and ‘*genic*’ from transgenic; rg). The original publication describes the generation of retrogenic mice expressing either the OTI- or OTII-OVA-specific TCR. Holst and colleagues detected frequencies of OTI^+^- or OTII^+^-T cells in the retrogenic mice that were similar to the one observed in respective OTI or OTII transgenic control animals [4], [5]. Subsequently, several other groups published the generation of mice expressing retrogenic TCRs, e.g. a MOG-peptide specific TCR [7]. Altogether, 64 different TCR retrogenic mice were generated in the past seven years [8]. In striking contrast, not a single BCRrg mouse has been published to date.

The generation of retrogenic mice offers several potential advantages compared with the generation of transgenic mice. First, retrogenic mice can be generated using any background strain. Second, the generation of retrogenic mice is faster than the generation of transgenic mice, since there is no need to backcross the retrogenic mice. Third, multiple proteins can be analysed simultaneously.

However, retrogenic mice cannot be propagated by breeding, because there is no germline transduction and the analysis is limited to the hematopoietic system [4].

Another major advantage of the retrogenic approach is the usage of so-called 2A peptides to link two or more target proteins instead of an IRES. Mechanistically, the 2A sequence induces the “skipping” of the ribosome thereby preventing it from covalently linking newly inserted amino acids and letting it continue translation. Therefore, 2A sequences are referred to as CHYSEL (*cis*-acting hydrolase element) sequences. This allows for the stoichiometric expression of the spliced proteins, which is crucial for heterodimeric molecules such as the BCR. Although these 2A like sequences were first discovered in +ssRNA and dsRNA viruses, the ribosomal “skipping” functions also *in vitro* and *in vivo* in all tested eukaryotic systems [9]–[12].

To demonstrate that the generation of BCR retrogenic mice is feasible per se, we chose the well-characterised Hen-Egg-Lysozyme (HEL)-specific BCR, MD4. A MD4 BCR-transgenic line was generated in the 1980s by Goodnow and co-workers and was used as control in our experiments [1].

We show for the first time the expression of a recombinant, membrane IgM-BCR *in vitro* using the pro-B cell line R5B, which is deficient for endogenous Ig chains. We also detected the recombinant αHEL IgM-BCR intracellularly when analysing these retrogenic mice, but to our surprise, we failed to demonstrate the surface expression of the recombinant αHEL IgM-BCR *in vivo*.

## Materials and Methods

### Mice

*Rag*2^-/-^ [13], OTII TCR transgenic [14] and C57BL/6 wt mice were bred and maintained under SPF conditions at the animal facility of the University Hospital Jena. HEL-IgM-BCR transgenic mice (MD4) [1] were kindly provided by R.J. Cornall (Oxford, UK).

### Ethics statement

All animal experiments were approved by the appropriate governmental authority (Thüringer Landesamt für Lebensmittelsicherheit und Verbraucherschutz; Registered Number 02–038/06) and conducted in accordance with institutional and state guidelines.

### Injection of 5-Fluorouracil

Donor mice were injected with 0.15 mg/g bodyweight 5-Fluorouracil (5-FU) intraperitoneally to induce proliferation of hematopoietic stem cells. After 72 hrs, bone marrow was isolated from femur and tibiae (see below).

### Irradiation of recipient mice

*Rag*2^-/-^ (4.5 Gy) or C57BL/6 wildtype (9 Gy) recipient mice were irradiated at the Leibniz Institute for Age Research/Fritz-Lipmann-Institute (FLI), Jena, using a *Gammacell40 Exactor* (Caesium source). Drinking water was supplemented with Sulfamethoxazol/Trimethoprim (Cotrim, 40 mg/ml; changed twice a week; Hexal, Holzkirchen, Germany) before irradiation and after reconstitution.

### Cloning of HEL-Igμ_m_ BCR

**Figure 1:**
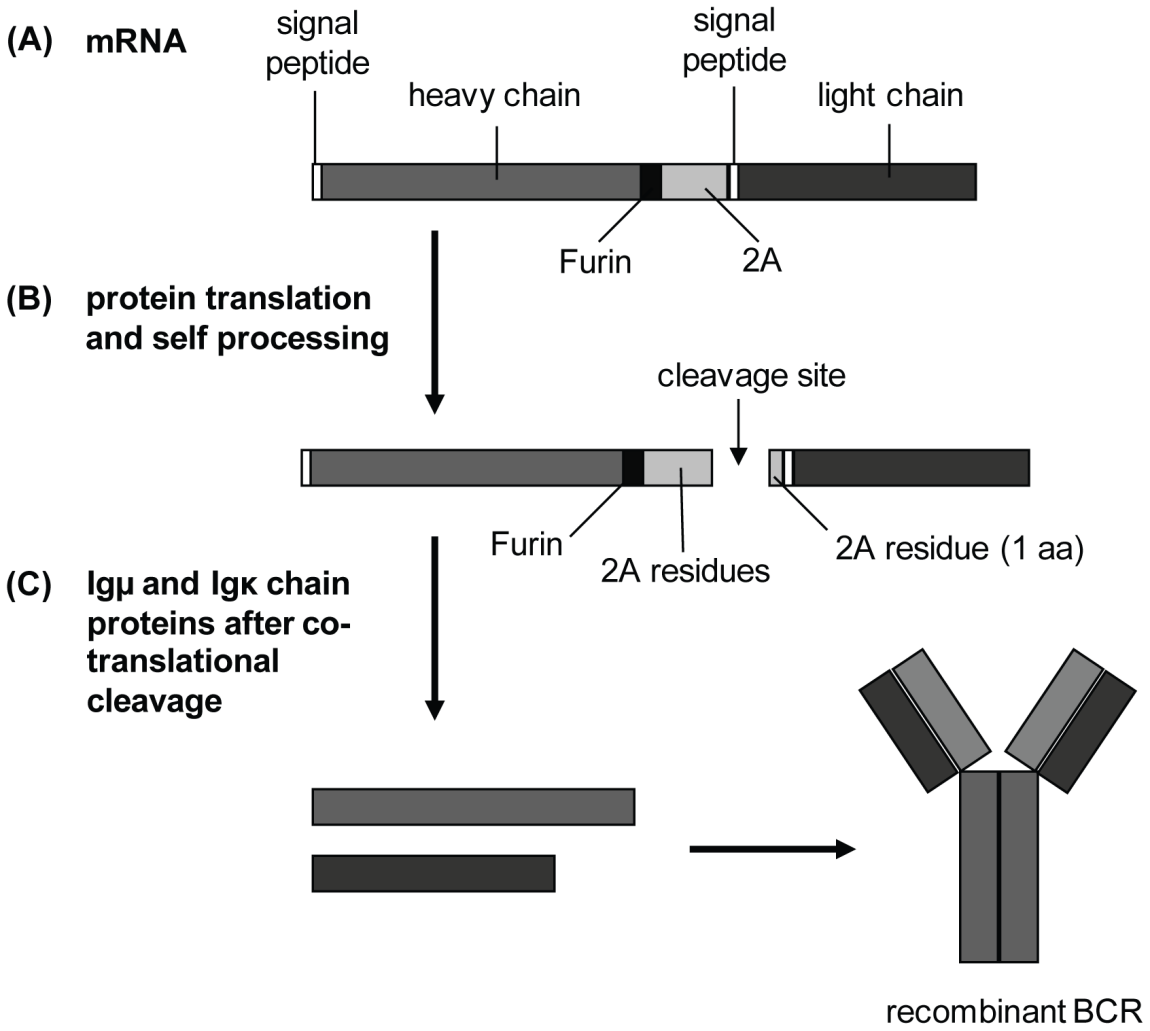
Full-length recombinant HEL-IgM-BCR expression cassette. (A) The HEL-specific Ig chains are linked by the Furin–FMDV-2A self-cleaving sequences. Both Ig chains have a signal peptide sequence, but only the downstream Igκ chain possesses a stop codon. (B) During translation, the first cleavage event occurs at the FMDV-2A peptide sequence; the 2A peptide remains attached as remnant to the C-terminus of the upstream Igμ chain. (C) A second cleavage event, initiated at the Furin site, finally yields both Ig chains without further attachments that can be assembled to build the HEL-IgM-BCR.

Total RNA from sorted transgenic HEL-Igμ^a+^ B cells was isolated using the High Pure RNA Isolation Kit (Roche Applied Science, Penzberg, Germany), reverse transcribed using Oligo(dT) and cDNA was subsequently used as template for cloning of the HEL-IgH and IgL chains. Full length sequence information for HEL-IgH and IgL chain genes were obtained through 5′ and 3′RACE (GeneRACER Life Technologies, Darmstadt, Germany). Using the below mentioned oligos, the FMDV-2A peptide sequence as well as restriction sites were added to the full length sequence clones for either HEL-specific Igμ as well as Igκ chain: forw_Igμ: 5′ GGGACCGGTGCCGCCACCATGATGGTGTTAAGTCTTCTGTAC; rev_Igμ: 5′GCC GGCAAGCTTCAGCAGGTCGAAGTTCAGGGTCTGCTTCACGGGGGCCCGCCGCCGCCGTTTCACCTTGAACAGGGTGACG; forw_Igκ: 5′CTGCTGAAGCTTGCCGGC GACGTGGAGAGCAACCCCGGCCCCATGGTTTTCACACCTCAGATACTT; rev_Igκ: 5′TCCCCGCGGGGACTAACACTCATTCCTGTTGAAGCT. Oligos were synthesized by BioTeZ (Berlin, Germany). Sequences of selected clones were analysed (Agowa/LGC Genomics, Berlin, Germany) at appropriate check points. The recombinant construct was subcloned into the retroviral based target vector (pRMYs-eGFP) via the restriction sites AgeI and SacII (see **Fig. 1** for detailed structure of the recombinant HEL-Igμ construct).

### Transfections

CHO-K1 cells were grown in DMEM (PAA Laboratories/GE Healthcare, Freiburg, Germany) supplemented with 10% FCS, 100 mM Hepes, 20 U/ml Penicillin, 0.1 mg/ml Streptomycin, 50 µM 2-Mercaptoethanol. For transfection, cells were seeded at 1.5×10^5^ cells/ml in RPMI (PAA Laboratories/GE Healthcare, Freiburg) w/o antibiotics. After 24 hrs, cells were transfected using Lipofectamine2000 transfection reagent (Life technologies, Darmstadt) and incubated for further 24 hrs. Transfection efficiency was determined as% GFP^+^ using FACS.

### Western blot

Cells were lysed with lysis buffer (20 mM Hepes, 2.5 mM MgCl_2_, 1% Triton X-100, 100 mM EGTA, 1 M β-Glycerophosphat, 100 mM ortho-Vanadat, 1 M DTT, 10 mg/ml Aprotinin, 1 mg/ml Leupeptin). Protein extracts were separated on 10% SDS-Laemmli gels and transferred by electroblotting onto nitrocellulose membranes. Membranes were blocked with BSA and incubated with primary antibody (goat anti-mouse Igμ, goat anti-mouse Igκ, both Santa Cruz Biotechnology, Santa Cruz, USA). Membranes were washed in 0.1% Tween/TBS and incubated with the HRP-conjugated secondary Ab (donkey anti-goat-IgG-HRP; Santa Cruz Biotechnology). Detection was performed using ECL reagent (Pierce).

### Producer cell generation and determination of viral titers

PhoenixEco cells [15] were grown in DMEM supplemented with 10% FCS, 100 mM Hepes, 20 U/ml Penicillin, 0.1 mg/ml Streptomycin, 50 µM 2-Mercaptoethanol. One day before transfection, cells were seeded at 4×10^6^ cells per 100 mm petri dish. Using calcium phosphate precipitation (2.5 M CaCl_2_, 2xHBS, 25 mM Chloroquine), cells were transfected with 20 µg plasmid DNA. Six to eight hours post-transfection, media was replaced. After 2 days, virus-containing supernatants were collected, sterile-filtered (0.45 µm) and kept at −80°C until further usage. Percentage of GFP^+^ cells was analysed using FACS.

For determination of viral titers, NIH/3T3 cells were seeded at 2×10^4^ cells/ml. At the next day, the cell culture media was removed and cells were centrifuged for 3.5 hrs at 33°C and 3300 rpm with virus-containing supernatants. To allow better transduction of the cells, polybrene (2 µg/ml, Sigma-Aldrich, Taufkirchen) was added. After 24 hrs the percentage of GFP^+^ cells was determined by flow cytometry. Viral supernatants with>45% GFP^+^ were used for infection of murine donor stem cells.

### Maintenance and transduction of cell lines

The WEHI-231 cell line, expressing surface Igμ [16], as well as the BCR-deficient pro-B cell line R5B [17] were maintained in RPMI supplemented with 10% FCS, 100 mM Hepes, 20 U/ml Penicillin, 0.1 mg/ml Streptomycin, 50 µM 2-Mercaptoethanol. For transduction of the R5B cell line, 5×10^5^ cells were centrifuged with virus-containing supernatant plus Polybrene (4 µg/ml) for 3.5 hrs at 3300 rpm and 33°C. After centrifugation, fresh media was added and cells were incubated for 48 hrs at 37°C and 5% CO_2_. After 2 days, cells were analysed for the surface expression of Igμ and Igκ as well as for the transduction rate (% GFP^+^) using flow cytometry.

The GP+E86 OTII producer cells used for the generation of OTII TCR retrogenic mice were kindly provided by D.A.A. Vignali (Memphis, Tennessee, USA) and maintained in DMEM supplemented with 10% FCS, 100 mM Hepes, 20 U/ml Penicillin, 0.1 mg/ml Streptomycin, 50 µM 2-Mercaptoethanol 2 mM L-glutamine, 1 mM sodium pyruvate and 100 mM MEM nonessential amino acids [4].

### Generation of TCR retrogenic mice

OTII TCR retrogenic mice were generated according to the protocol published by Holst et al. [4]. Therefore, donor mice were injected with 0.15 mg per g body weight 5-Fluorouracil. After 48 hrs, bone marrow was extracted from femur and tibiae and cultured in cDMEM (20% FCS) supplemented with 20 ng/ml rmIL-3, 50 ng/ml rmIL-6 and 50 ng/ml SCF. GP+E86 OTII producer cells were irradiated with 12 Gy 24 hrs after the donor BM cells had been isolated. Transduction was allowed for 48 hrs. A minimum of 4×10^6^ cells was used for reconstitution of lethally irradiated wildtype recipient mice.

### Isolation and culture of mouse bone marrow-stem cells

Bone marrow was flushed from femur and tibiae of donor mice using a 23xG needle. Cells were seeded at 1.5–2×10^6^ cells/ml and incubated for 48–72 hrs at 37°C +5% CO_2_ prior to infection. For the generation of BCR rg mice, cell culture media was further supplemented with 20 ng/ml IL-3 (BPV supernatant [18],), 50 ng/ml rmIL-6, 10 ng/ml rmIL-7, 20 ng/ml SCF and 100 ng/ml Flt3-L (all from Miltenyi Biotec, Bergisch-Gladbach, Germany).

### Transduction of murine bone marrow-stem cells

For transduction of the donor cells, cell culture plates were coated overnight with RetroNectin (40 µg/ml; TaKaRa Bio, Saint-Germain-en-Laye, France) at 4°C. The next day, the plates were washed twice with PBS and blocked with 2% BSA/PBS. Virus-containing supernatants were added and plates were centrifuged twice for 2 hrs at 2000×*g* and 32°C, while the supernatants were replaced after the first 2 hrs of centrifugation. Donor BM cells were placed onto these virus-loaded plates at 2×10^6^ cells/ml and incubated at 37°C+5% CO_2_. After 24 hrs, cells were harvested and intensively washed. Transduction efficiency (% GFP^+^ cells) was determined using flow cytometry. Transduced cells were resuspended in 2% FCS/PBS and used for reconstitution of irradiated recipient mice (minimum 4×10^6^ cells/mouse).

### Flow cytometry and cell sorting

Transduced cell lines (WEHI-231, R5B) or single-cell suspensions (prepared from mouse spleens, lymph nodes and bone marrow) wer eincubated with anti-CD16/32 (2.4G2/75; 10 µg/mL) and rat IgG (10 µg/mL; Dianova, Hamburg, Germany) to prevent unspecific binding. Cells were stained with anti-Igμ^a^-biotin (clone DS-1), anti-Igμ^b^-PE (clone AF 6–78; both BD Biosciences, Heidelberg, Germany), anti-mouse IgM-Cy5 (μ-chain specific), anti-mouse Igκ-PE (both Southern Biotech/Biozol, Eching, Germany), anti-mouse CD19-Alexa Fluor 647 (clone 1D3; eBioscience, Frankfurt/Main, Germany). Streptavidin-APC-eFluo780 (eBioscience) was used as secondary antibody; Hen-Egg-Lysozyme was coupled to Alexa Fluor 647 (kindly provided by René Riedel, Deutsches Rheumaforschungszentrum Berlin, Berlin, Germany). For intracellular stainings, cells were fixed with 2% PFA for 20 min on ice and subsequently permeabilised by washing with saponin-containing buffer. Again, unspecific binding of antibody was blocked by incubation with anti-CD16/32 (2.4G2/75; 10 µg/mL) and rat IgG (10 µg/mL; see above). 1 000 000 events were acquired for each sample using a LSRII cell cytometer (BD Biosciences). Data were acquired using the DiVa software; data analysis was performed using FlowJo software (TreeStar). For the isolation of HEL-specific, Igμ^a+^ B cells, splenocytes of transgenic HEL-IgM-BCR mice were first depleted of T cells by magnetic cell sorting (AutoMACS; Miltenyi Biotec) using CD90-Microbeads (mouse, Miltenyi Biotec). Thereafter CD90-negative cells were further stained with an allotype-specific antibody (anti-Igμ^a^-FITC, clone DS-1; BD Biosciences) and FACS-sorted (ARIA, BD Biosciences).

## Results

### Cloning of the recombinant HEL-IgM B-cell receptor

To establish the generation of BCR retrogenic mice we decided to use the Hen-Egg-Lysozyme (HEL)-specific membrane form of μ heavy chain (Igμ) and the κL chain (Igκ) of the BCR MD4. A corresponding transgenic line, expressing HEL-specific IgM as well as IgD antigen receptors, was generated by Goodnow and co-workers. The MD4 μ and δ genes were derived from BALB/c mice (IgH^a^ allotype) and can, therefore, be distinguished from the endogenous C57BL/6 BCRs (IgH^b^ allotype) [1]. We purified the HEL-specific B lymphocytes from transgenic MD4 splenocytes by FACS (purity>95%), using an allotype-specific antibody (anti-Igμ^a^) (see Figure S1). Total RNA was isolated from these cells, reverse transcribed and used as template. First, we obtained full length sequence information for both Ig chains by performing 5′ and 3′ RACE. A 2A-peptide sequence was added to the full length cDNA sequences of the HEL-specific μ- and κ-light chain by PCR. The recombinant construct was generated through ligation employing the introduced restriction sites. The expression cassette comprises 5′LTR–HEL-Igμ–furin–2A peptide–Igκ–IRES–eGFP–3′LTR (Fig. 1).

To link the two HEL-specific Ig chains (resulting in the recombinant BCR) we chose the Foot-and-Mouth-Disease-Virus- (FMDV-) derived 2A peptide sequence. We used this particular 2A peptide sequence (APVKQTLNFDLLKLAGDVESNPGP) because cleavage efficiencies of>90% were reported using this 2A peptide sequence [19]–[21]. The DNA sequence of selected clones was confirmed at appropriate time points. One clone, carrying the recombinant HEL-IgM-BCR, was selected for all further experiments. Initially, we also included a different construct (with the Igκ-chain being the upstream protein). However, we did not succeed in generating the recombinant construct for this variant of the HEL-Igμ BCR due to the formation of tertiary structures. As a consequence, we performed all subsequent experiments with the expression cassette shown in Fig. 1.

### Verification of the FMDV-2A peptide-mediated cleavage *in vitro*

The 2A peptide-mediated cleavage event occurs during the translation process. Thereafter, the 2A peptide remains attached as remnant to the polypeptide encoded by the sequence upstream of the 2A peptide. To date, no deleterious effects of the remaining 2A peptide have been observed in retrogenic mice [8]. Here, the remnant is attached to the C-terminus of the membrane form of Igμ. Since the correct sequence of the C-terminus is essential for surface expression of the BCR, we decided to include also a Furin cleavage site (RRRR) [22]–[24] upstream of the FMDV-2A to ensure removal of 2A peptide remnant and thus to obtain a more native Igμ chain by post-translational processing (Fig. 1).

To verify the cleavage efficiency of the FMDV-2A peptide as well as the upstream Furin-cleavage site, we transfected Chinese Hamster Ovary cells (CHO-K1) with the retroviral construct and analysed lysates by western blot (Fig. S2). Lysates of either empty vector-transfected cells or of hybridomas expressing either Igμ or Igκ [25], [26] served as controls. Using anti-mouse Igμ- and Igκ-specific antibodies, specific products for both Ig chains (∼28 kDa for Igκ; ∼70 kDa for Igμ) could be detected in the lysates of HEL-IgM-BCR transfected cells, indicating cleavage of the recombinant protein.

However, with both primary antibodies another high-molecular-weight band was detected. Its apparent molecular weight of ∼100 kDa corresponds to the expected size of the uncleaved, recombinant protein providing evidence for incomplete processing. Since the intensities of the Igμ- and the Igκ-specific bands were similar to that of the uncleaved product detected with anti-Igμ and anti-Igκ, respectively, we concluded that the amounts of cleaved versus uncleaved protein were nearly equal. Hence we proceeded with the *in vitro* surface expression of the recombinant BCR.

### Expression of the recombinant HEL-IgM-BCR in a BCR-deficient cell line

**Figure 2:**
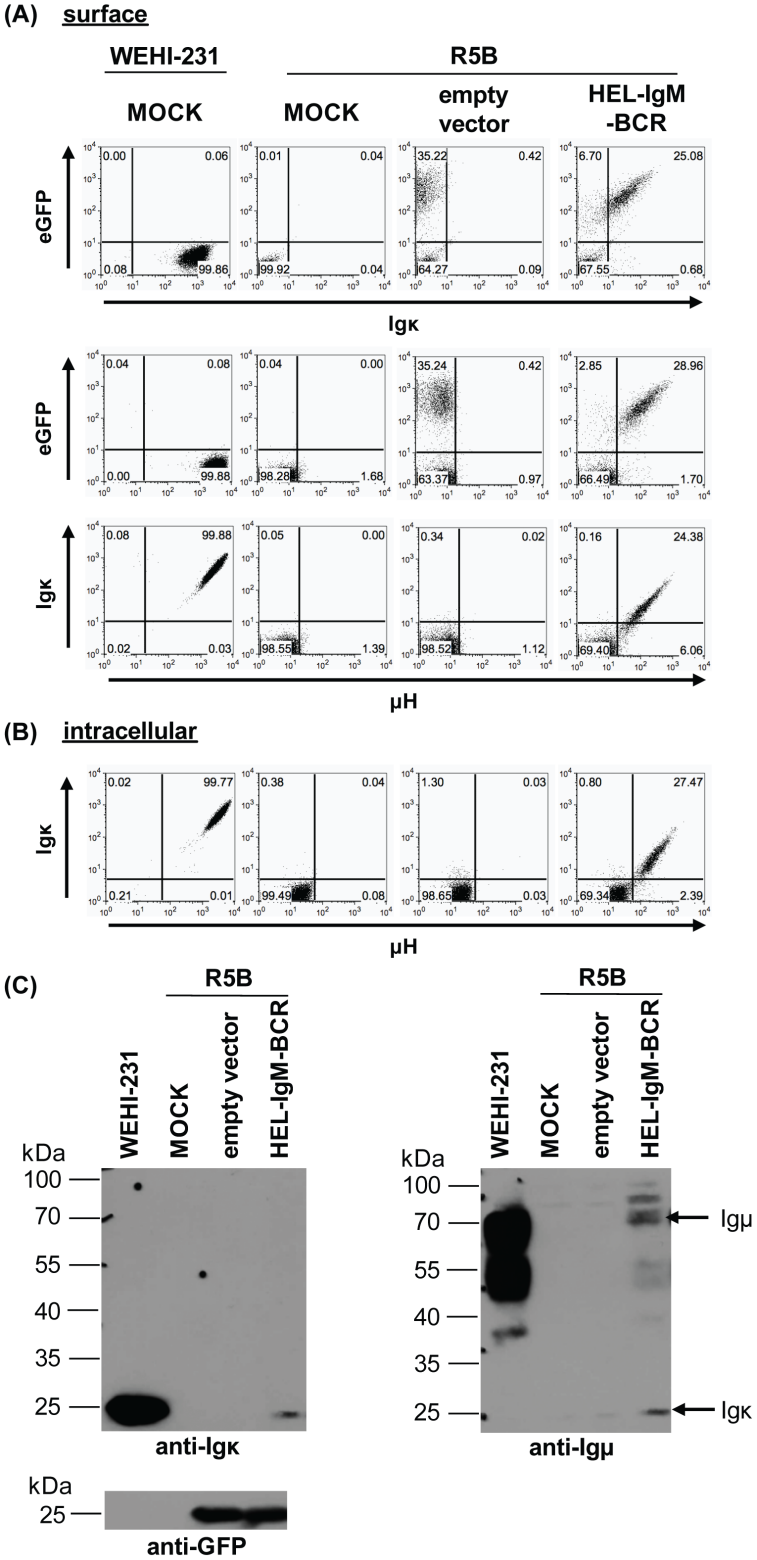
*In vitro* surface expression of the recombinant HEL-IgM-BCR. The pro-B cell line R5B, deficient for endogenous BCR, was infected with virus-containing supernatant encoding for the recombinant HEL-specific IgM-BCR. 24 h post-infection, cells were either analysed by flow-cytometry or western blot analysis. The WEHI-231 B-cell lymphoma served as positive control. Staining for Igμ and Igκ showed surface (A) as well as intracellular expression (B) of the recombinant BCR. Numbers in quadrants indicate the percentage of cells expressing the respective marker. (C) Lysates of infected R5B cells were analysed for κ- and μH-chain expression. Expression of the Ig chains was only detected in lysates of cells infected with the recombinant HEL-IgM-BCR. Incubation with an anti-GFP antibody was used as internal control. Results are representative of three independent experiments.

To examine the production and assembly of the recombinant HEL-Ig_M_-BCR *in vitro*, we transduced the IL-7-dependent pro-B cell line R5B (*Rag2* ^-/-^) [17], [27] with virus-containing supernatant. R5B cells do not express endogenous Ig chains, but do express the surrogate light chain. Supernatants of either empty vector transfected or MOCK-transfected cells as well as the IgM(κ)-producing cell line WEHI-231 [16] were used as controls in this experiment. 48 hours after the transduction, the cells were stained with either fluorochrome-conjugated Igμ- or Igκ-specific antisera and analysed by flow cytometry (Fig. 2A). As expected, neither Igμ nor Igκ could be detected on the cell surface of the non-transduced or empty vector transduced samples. Specific staining for Igμ as well as Igκ was shown for the WEHI-231 control. Furthermore, all cells that were transduced with retrovirus encoding for the recombinant BCR (and thus were GFP^+^), expressed the recombinant BCR (∼28% Igκ^+^Igμ^+^). The two Ig chains were always co-expressed. The Ig chains could also be detected intracellularly (Fig. 2B). Again, co-expression of the Igμ and Igκ chain was observed for cells that were transduced with virus encoding for the recombinant BCR (∼27% Igκ^+^Igμ^+^). Interestingly, the frequencies of μH^+^Igκ^+^ cells were equal for surface as well as for intracellular stainings, suggesting that all transduced cells transported the recombinant BCR to their cell surface.

Stoichiometric expression of the two Ig chains could also be shown at the protein level by western blot analysis. Using anti-mouse Igμ- and Igκ-specific antibodies, specific products for both Ig chains (∼70 kDa for μH, ∼28 kDa for Igκ) could be detected in the lysates of cells infected with supernatant encoding for the recombinant BCR but not in the non-transduced cells and cells transduced with empty vector controls (Fig. 2C).

### Generation of retrogenic mice expressing HEL-specific IgM-BCRs

Whereas the generation of many different TCR retrogenic mice has been published; no BCR rg mice have been published to date. Therefore, we decided first to generate TCR rg mice to establish the procedure in our lab. We generated OTII TCR (TCRVα2–P2A–TCRVβ5–IRES-eGFP) rg mice according to the publication of Holst et al. and compared them with the corresponding classical OTII TCR transgenic mice [5]. Flow cytometric analysis of the TCR retrogenic mice at 8 weeks post-reconstitution showed that the ovalbumin-specific OTII TCR was expressed in all lymphatic organs analysed albeit with a lower frequency as compared to the transgenic controls (Fig. S3). Of note, already Holst et al. described in their original publication in 2006 lower frequencies (numbers) in the retrogenic as compared to the transgenic system [4].

Having established the generation of TCR retrogenic mice, we started to modify the culture conditions of the donor cells for the differentiation of B cells rather than T cells (see Materials and Methods section). An adapted protocol was used to transduce donor cells (as compared to [4]) giving better transduction efficiencies in our hands. Figure S4 exemplary shows the flow cytometric analysis of HEL-IgM-BCR transduced donor stem cells at either 24 hours or 5 days post-transduction. Transduction rate was ∼6% (as expressed by GFP^+^ cells) at 24 hrs post-infection. The percentage of GFP^+^ cells was even increased after further 4 days in culture along with the percentage of GFP^+^CD19^+^ cells, indicating the stem cells would at least partly develop into the B cell lineage. Transduced cells were subsequently used to reconstitute lethally irradiated wildtype recipients.

**Figure 3:**
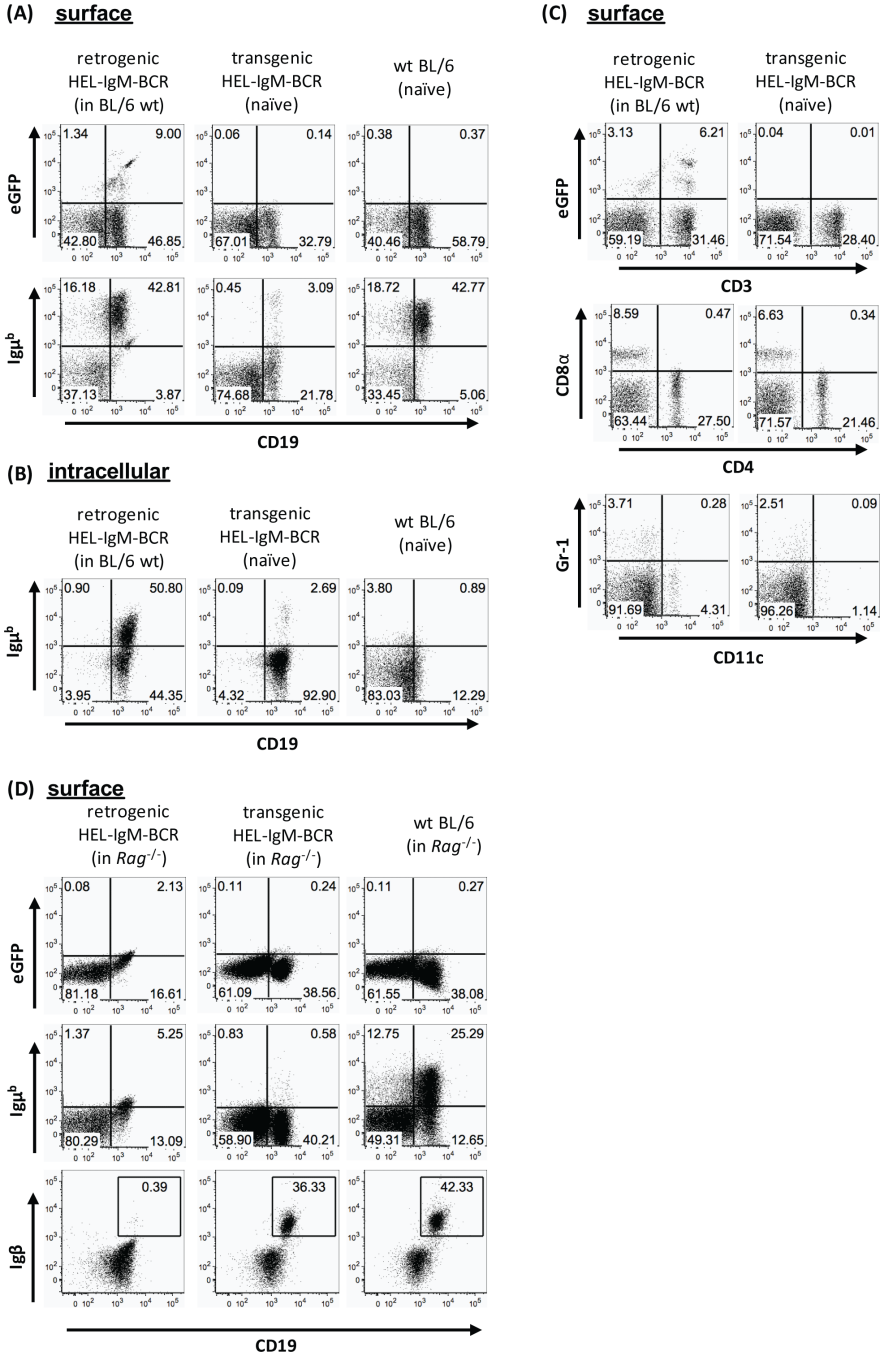
Analysis of HEL-IgM retrogenic mice showed only weak expression of the retrogene. Retrogenic mice were generated as described in the Materials and Methods section. Analysis was performed 6- to 8-weeks post-reconstitution. Single cell suspensions of spleen and lymph nodes were stained with fluorochrome-conjugated antibodies and analysed by flow cytometry. Transgenic HEL-IgM-BCR as well as wildtype C57BL/6 mice served as controls. Results for splenocytes are displayed. (A) Representative surface staining for CD19 and Igμ^b^. Transduced cells are GFP^+^. Numbers in quadrants indicate the percentage of cells expressing the respective marker. Dead cells were excluded from the analysis. (B) Intracellular staining for the same parameters as in (A). Cells are gated on CD19^+^ cells. (C) Surface staining for surface markers other than B cells (CD3, CD4, CD8, CD11c, Gr-1). (D) *Rag*2^-/-^ mice served as donors and recipients instead of C57BL/6 wt mice. Flow cytometric analysis for same parameters as in (A) as well as for Igβ. Results shown are representative of two independent experiments.

Starting 2 weeks post-reconstitution, mice were bled regularly to track the repopulation of the immune system (i.e. B cells), thereby identifying the optimal time point for analysis. Around 8 weeks post-reconstitution, IgM-BCR retrogenic mice were sacrificed, single cell suspensions of spleen, lymph node, bone marrow as well as blood were prepared, stained with fluorochrome-conjugated antibodies against B cell marker and analysed by flow cytometry. Figure 3 shows exemplary results for splenocytes from HEL-IgM-BCR retrogenic mice. The percentage of GFP^+^ (and therefore transduced) cells was ∼10%. Of note, only those transduced cells will express the retrogenic BCR on their surface that co-express the signaling components of the BCR complex (Igα and Igβ) and therefore will develop into B cells. With the help of the TCR retrogenic mice it was already shown that only cells co-expressing the TCR-co-complex molecule CD3 would express the rg TCR on their cell surface [4].

Analysis of the HEL-IgM-BCR retrogenic mice further revealed GFP^+^ cells expressing the endogenous Igμ of b allotype at levels comparable to the wildtype control group (42.81% and 42.77% CD19^+^Igμ^b+^ respectively). However, surface staining demonstrated no expression of the retrogene, since no specific binding of the antigen HEL coupled to a fluorochrome was observed. Specific HEL-binding was observed, as expected in the HEL-IgM-BCR transgenic mice that were carried as control (Figure S5A, middle panel). Of importance, the fluorochrome-conjugated HEL used in these experiments exhibited unspecific binding to a certain degree. Nevertheless, the BCR signaling components Igα and Igβ were expressed by all groups analysed (data not shown).

In another experiment, we reconstituted irradiated wildtype mice with transgenic MD4 bone marrow cells (no retrovirally transduced cells were used in this experiment). Analysis performed at 6–8 weeks post-reconstitution showed that ∼13% of splenic as well as ∼6% of lymph node cells were positive for the transgene (by staining for CD19, Igμ^a^ and HEL; see Figure S6).

Whereas the retrogenic mice did not express the HEL-specific IgM-BCR on their surface in detectable amounts, intracellular staining revealed high expression of the retrogene; (Figure S5B). In contrast to HEL-BCR transgenic mice, B cells from the retrogenic mice expressed both intracellular HEL-IgM-BCR (with Igμ^a^), as shown by staining with the antigen HEL coupled to a fluorochrome and the endogenous Igμ (Igμ^b^; Figure 3B and Figure S5B). Surface staining for lineage markers other than B cells revealed no major differences between the analysed groups (Figure 3C). Solely the percentages of CD4^+^ and CD8^+^ (and therefore also CD3^+^) T cells were slightly increased in the retrogenic compared with the transgenic mice (27.50% to 21.46%, 8.59% to 6.63% and 31.46% to 28.40%).

In a final attempt, we reconstituted *Rag*2^-/-^ mice to rule out possible expulsion by endogenous, Igμ^b+^ cells. Again, we were not able to detect the retrogenic HEL-IgM-BCR in the analysed mice, although we could identify the transgenic HEL-IgM-BCR in mice reconstituted with transgenic bone marrow cells (as shown in Figure S5C). Furthermore, by staining for the BCR signaling components Igα and Igβ we could not show expression of these molecules for the HEL-IgM-BCR retrogenic mice but for both the transgenic and wildtype reconstituted controls that were carried in this experiment (figure 3D). However, the frequency of retrogenically transduced cells in this experiment was low (∼2% GFP^+^). This should be taken into consideration when interpreting this set of data.

In summary, we were able to generate mice expressing the retrogenic BCR albeit only intracellularly. However, the BCR was not transported to the cell surface.

## Discussion

The generation of retrogenic mice was originally described in 2006 by the group of D.A.A. Vignali for TCRs. They described retrovirally-mediated stem cell gene transfer to express TCRα and TCRβ chains from a 2A peptide-linked multicistronic retroviral vector in mice [4], [5]. Since then several other groups successfully generated TCR retrogenic mice (for examples see ref. [8]). The number of publications describing the generation of TCR retrogenic mice has exceeded the number of 60, with the retrogenic TCRs being specific for model antigens (as OVA, male antigen) or with the TCR's specificity being relevant during autoimmunity (e.g. insulin, MOG) or host defence (influenza). In contrast and surprisingly, we are unaware of a single publication on the generation of BCR retrogenic mice. We reasoned that this approach should be amenable to the production of BCR retrogenic mice expressing IgH and IgL chains from a 2A peptide-linked multicistronic retroviral vector. Among the early publications on retrogenic technology one also speculated that generating BCR retrogenic mice should be possible [6]. After having produced TCR-retrogenic mice (using the well characterised OTII TCR that recognises an ovalbumine peptide presented by I-A^b^) we set out to produce BCR retrogenic mice.

As a model BCR we chose the well characterized Hen-Egg-Lysozyme-specific BCR MD4 [1]–[3]. Using the *Rag*-deficient pro-B cell line R5B [17] we were able to show the surface expression of the retrogenic HEL-IgM-BCR *in vitro*. However flow cytometric analyses of our BCR retrogenic C57BL/6 mice revealed that the HEL-IgM-BCR was expressed exclusively intracellularly but not on the cell surface. To rule out competition by endogenous BCRs we used *Rag*2^-/-^ mice. Even in these mice the retrogenic HEL-IgM-BCR was detectable only intracellularly (data not shown) but not as membrane protein suggesting that the processing of the BCR for transport to the cell surface in R5B cells differs from the requirements *in vivo*. By contrast, although highest *in vitro* cleavage efficacies were shown for 2A peptide derived from Foot-and-Mouth-Disease-Virus [19]–[21], Kim et al. showed highest *in vivo* cleavage efficacies for porcine teschovirus-derived 2A sequence [28]. Therefore, it is likely that the cleavage events mediated by the FMDV-derived 2A peptide or furin were improper *in vivo*, thus preventing the correct insertion of the Igμ chain into the cell membrane. At the same time, this could also explain why a transgenic BCR but not a retrogenic BCR is transported to and expressed at the cell surface.

In general, the surface expression of a BCR requires the expression of the BCR signaling components Igα and Igβ in addition to both Ig chains. All four components are required to assemble a transport-competent BCR [29]. Using fluorochrome-conjugated antibodies specific for Igα and Igβ we were able to show the expression of these molecules on the surface of the B lymphocytes of the analysed retrogenic mice. Consequently, the failed surface expression of the retrogenic anti-HEL-IgM-BCR cannot be accounted for the lack of expression of Igα and Igβ. Finally, the GFP expression is not representative of HEL-specific BCR but of transduced cells. Since bone marrow stem cells are used for the transduction, they may differentiate into cells other than B cells (e.g. T cells, as shown by GFP^+^CD3^+^ cells in Fig. 3C). This phenomenon was already described by Holst et al. as they detected GFP^+^ cells that were CD3^−^ and therefore did not express the retrogenic TCR and presumably represented cells other than T cells [4], [5].

One important point to consider in our efforts to produce BCR retrogenic mice is the seemingly low rate of transduction. In our experiments we detected transduced (and therefore GFP^+^) cells with a frequency of ∼10%. This is in line with the frequencies described by Holst et al. in their original publication on the generation of retrogenic mice [4]. Therefore, the low rate of transduction is unlikely to explain the lack of surface expression. Of note and as shown in figure S4, when we analyzed our transduced cells 5 days post-infection, the percentage of GFP^+^ along with the percentage of GFP^+^CD19^+^ cells was increased as compared to the 24 hrs post-infection time point arguing the stem cells would at least partly develop into the B cell lineage.Hypothetically, the lower frequency observed in our scenario could be due to the particular receptor used. Holst et al. reported that the results obtained with the retrogenic approach will vary depending on the specificity of the TCR to be investigated. However all retrogenically analysed TCRs were expressed [4]. Whereas we did not formally rule out this possibility for our BCR retrogenic system we consider it unlikely.

One final and important question is whether the failure to produce retrogenic mice with one specific BCR reflects a principle problem to express such a construct in B cells, or whether it is due technical or other problems particular to the BCR, the vector used or other non-generalisable factors. Whilst this cannot be answered definitively with one, two or several negative attempts, we know of one other group who used a different BCR and a different vector construct and also failed to produce retrogenic mice despite intensive efforts (F. du Pré and L.M. Sollid, U. Oslo, personal communication). The two groups' similarly negative results together with our positive results regarding the production of TCR rg mice, support the interpretation that the problem is general (for retrogenic BCRs) and not particular for one (or two) receptor constructs. Therefore, the failure to generate BCR retrogenic mice points towards an inherent and important difference between retrogenic BCR and TCR constructs.

In summary we used and optimised an approach that has proven successful for the generation of TCR retrogenic mice in several laboratories including ours to produce BCR retrogenic mice. Expression of the retrogenically encoded BCR was routinely detectable intracellularly. In contrast, surface expression of the retrogenic BCR was only achieved *in vitro* in the *Rag*-deficient pro-B cell line R5B but neither in *Rag*2^-/-^ mice nor in wild type mice *in vivo*.

Based on our results and the absence of any reports on BCR retrogenic mice in the literature we conclude that the retrogenic technology, while very useful to study other receptors including TCR, is not the obvious choice for expressing a BCR *in vivo*. Our findings are further corroborated by experiments performed by another group, who also failed in generating BCR retrogenic mice (M.F. du Pré and L.M. Sollid, University of Oslo, personal communication). Still, these combined negative results together with the absence of any reports on BCR retrogenic mice in the literature do not definitively rule out the potential use of BCR retrogenic expression.

## Supporting Information

Figure S1 **Isolation of MD4 transgenic B lymphocytes.**
(TIF)

Figure S2 **Verification of 2A peptide-mediated cleavage in CHO-K1 cells.**
(TIF)

Figure S3 **Comparison of OTII TCR rg and tg mice.**
(TIF)

Figure S4 **Transduction of donor BM cells.**
(TIF)

Figure S5 **Analysis o f HEL-IgM retrogenic mice showed only weak expression of the retrogene.**
(TIF)

Figure S6 **Reconstitution of irradicated wildtype mice with transgenic MD4 BM cells.**
(TIF)
