# Supplementary figures and images for: Towards the Generation of B-Cell Receptor Retrogenic Mice
Source: PLoS One. 2014 Oct 8;9(10):e109199. doi: 10.1371/journal.pone.0109199 (PMC4189916; doi:10.1371/journal.pone.0109199)

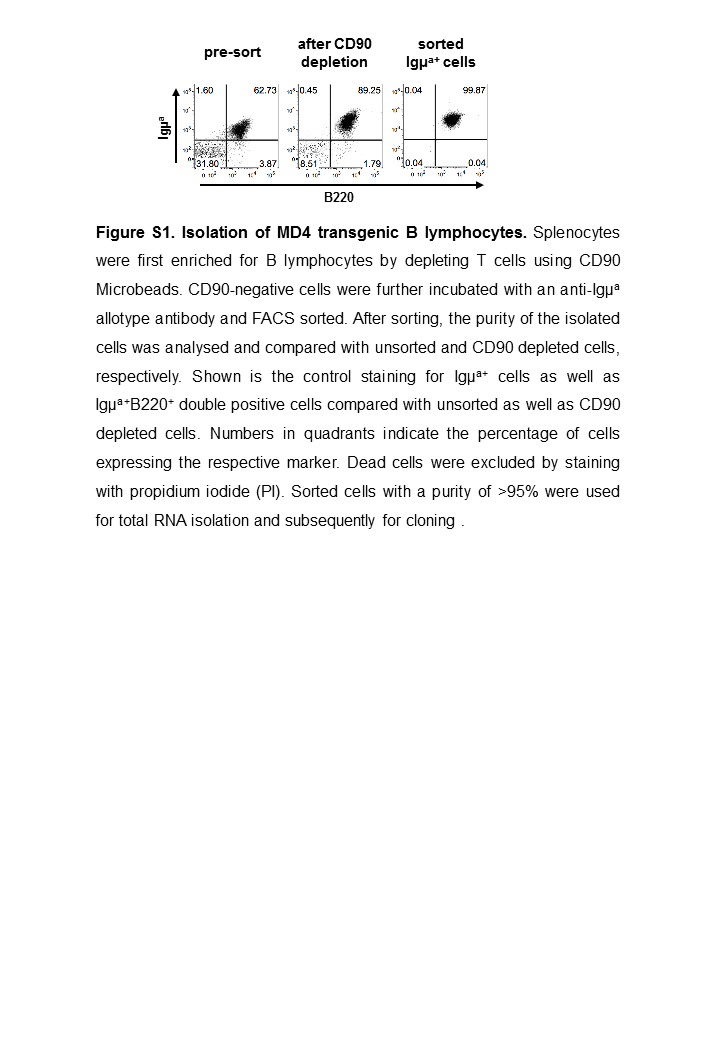

Supplement: Figure S1 — Isolation of MD4 transgenic B lymphocytes. (TIF) [file pone.0109199.s001.tif]

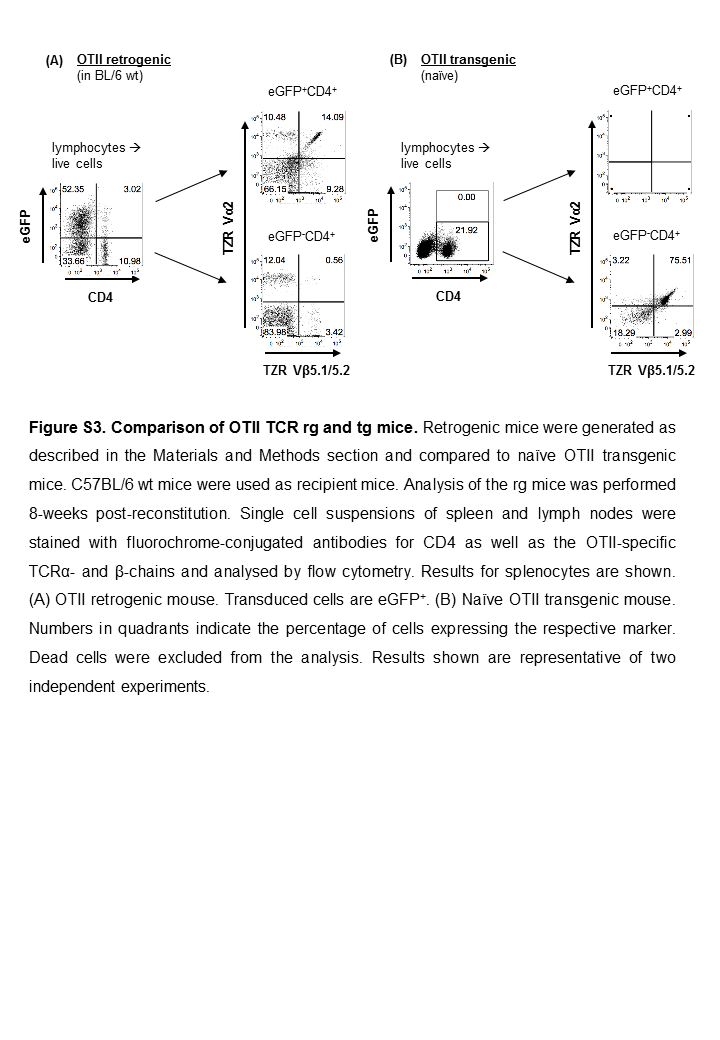

Supplement: Figure S3 — Comparison of OTII TCR rg and tg mice. (TIF) [file pone.0109199.s003.tif]

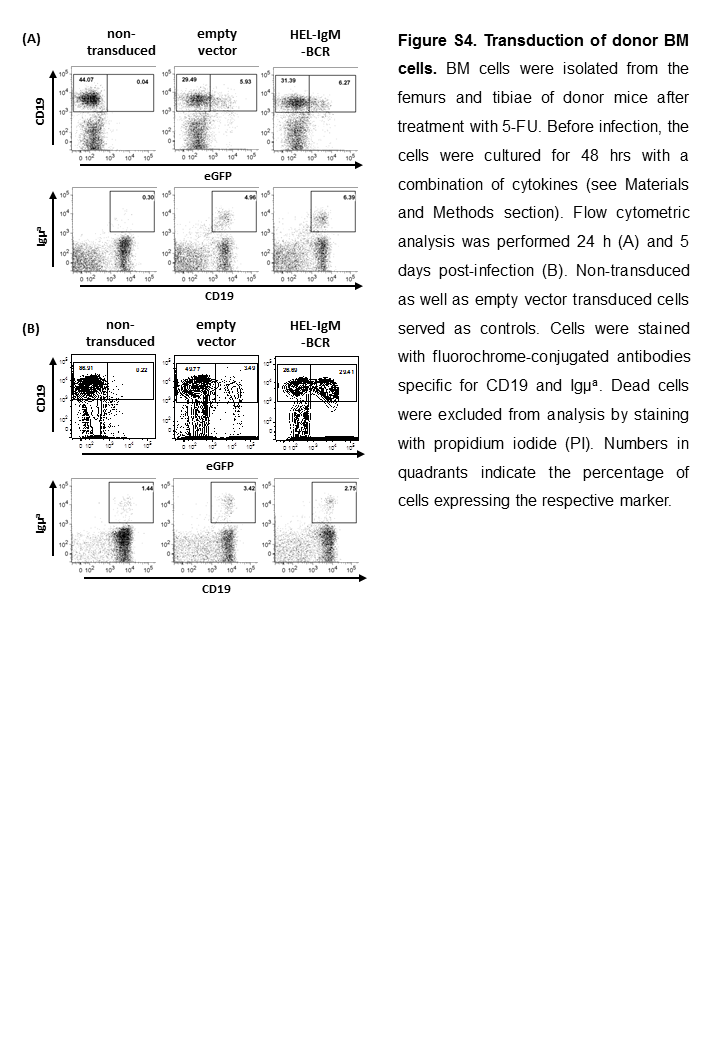

Supplement: Figure S4 — Transduction of donor BM cells. (TIF) [file pone.0109199.s004.tif]

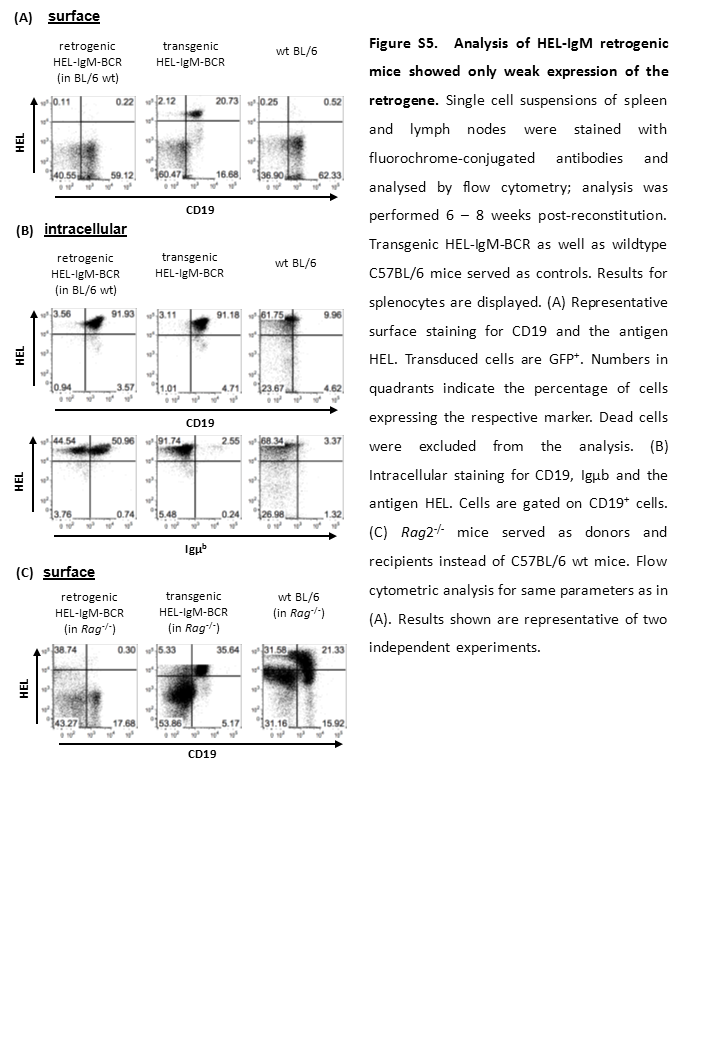

Supplement: Figure S5 — Analysis o f HEL-IgM retrogenic mice showed only weak expression of the retrogene. (TIF) [file pone.0109199.s005.tif]

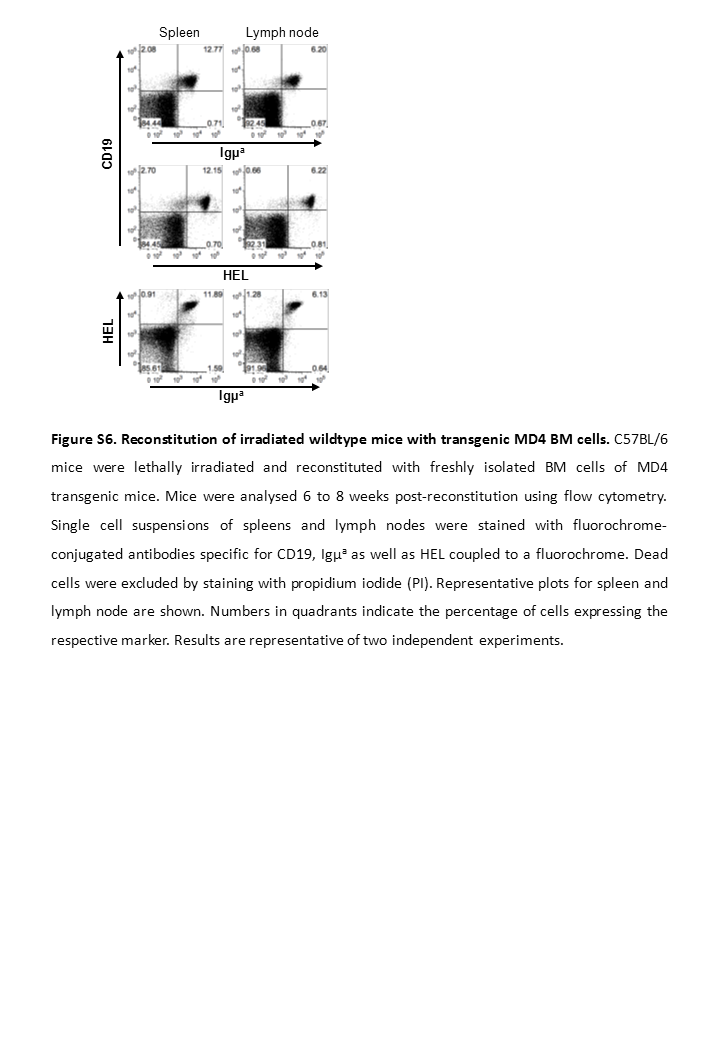

Supplement: Figure S6 — Reconstitution of irradicated wildtype mice with transgenic MD4 BM cells. (TIF) [file pone.0109199.s006.tif]
